# An algorithm for the use of anti-obesity medications

**DOI:** 10.1038/s41387-024-00278-2

**Published:** 2024-04-18

**Authors:** Fereshteh Dehghani, Mitra Ali Ahmadi, Marleigh Hefner, Gaurav Kudchadkar, Wasiuddin Najam, Masoud Nateqi, Md Abu Bakkar Siddik, Holli Booe, Nikhil V. Dhurandhar

**Affiliations:** 1 Department of Nutritional Sciences, Texas Tech University, Lubbock, TX USA; 2 Department of Epidemiology and Biostatistics, Indiana University School of Public Health-Bloomington, Bloomington, IN USA

**Keywords:** Therapeutics, Medical research

Reduced energy diet and increased physical activity form the cornerstone of medical management of obesity. However, long-term adherence to a restricted diet is highly challenging [1]. Anti-obesity medications (AOM) help to resist food cravings, reduce hunger, or increase satiety, thereby empowering individuals to adhere to a restricted diet and promote greater weight loss [2]. Therefore, AOM are needed, not as a substitute, but as a supplement to lifestyle modification efforts for weight management. The concept of pharmacological support for obesity treatment is similar to the use of medications for other chronic diseases with a behavioral component, such as diabetes or hypertension. Fortunately, several drug options are now available to treat hypertension or diabetes. And, in case of a suboptimal response of an individual to a drug, a health care provider (HCP) may choose a different drug or a different set of drugs. Similarly, if an individual is a poor responder to a particular AOM, that drug may still help another individual, and a different AOM may be more effective for the person who did not respond initially [3].

Often, the drug selection for treatment is based on many criteria, including accompanying comorbidities, prior response to medications, potential adverse events, and drug costs. For example, for diabetes treatment, secretagogues, mimetic, or sensitizers of insulin may be selected depending on the individual’s pathophysiology and circumstances. Similarly, AOM are comprised of medications with a range of target pathways, adverse event profiles, routes of administration, costs, and weight loss response [2].

However, AOM face additional challenges in recognition of their need for obesity management. While diabetes is widely recognized as a disease that needs aggressive medical attention, obesity may not be viewed by the patients or some HCPs as a serious disease that needs lifelong management, including pharmacotherapy. Furthermore, due to the refractory nature of obesity, resource intense treatment, and limited health insurance coverage, the window of opportunity to treat obesity is limited. Individuals with obesity who are less successful in an initial weight loss attempt are likely to abandon treatment and are less likely to initiate a new weight management attempt [4]. In addition, there are a limited number of Food and Drug Administration (FDA)-approved AOMs for long-term care. Therefore, a careful matching of AOM to an individual’s need is needed to maximize the chances of weight loss success. Hence, we propose an algorithm based on the indications of various FDA-approved AOM. First, a very brief description of AOM is merited.

## Drug description

### Glucagon-like peptide-1 (GLP-1) receptor agonist

This class of drugs, like Liraglutide and Semaglutide, mimics the action of glucagon-like peptide-1 and help regulate appetite. These drugs are intended for use by adults and children (≥12 years) with obesity or by adults with overweight (body mass index (BMI) ≥ 27) in the presence of weight-related comorbidities (Source: www.saxenda.com; www.wegovy.com).

### Glucose-dependent insulinotropic polypeptide (GIP) and GLP-1 receptor agonist

This medication (Tirzepatide), when combined with calorie-deficit diet and exercise, is helpful for managing weight in adult with obesity or overweight individuals who have weight-related comorbidities (Source: www.zepbound.lilly.com).

### Phentermine/Topiramate

Phentermine and Topiramate work together to control hunger and reduce cravings. This medication should be used in combination with a reduced-calorie diet and increased physical activity by patients aged 12 years and older with obesity or by adults with overweight (BMI ≥ 27) having weight-related comorbidities (Source: www.qsymia.com).

### Hydrogel (Plenity)

This hydrogel capsule works by expanding in the stomach, creating a feeling of fullness and usually taken before meals with water. Hydrogel is recommended to help with weight management in adults with overweight or obesity (BMI 25–40) in combination with diet and exercise (Source: www.myplenity.com).

### Naltrexone/Bupropion

Naltrexone and Bupropion work together to reduce hunger and control cravings and recommended in combination with lifestyle modifications in adults with obesity or overweight (BMI ≥ 27) and have weight-related medical conditions (Source: www.contrave.com).

### Setmelanotide

This medication is to be used only in adults and children 6 years of age and older who have obesity due to deficiency of POMC, PCSK1, or the leptin receptor (Source: www.imcivree.com).

### Orlistat

Orlistat inhibits fat absorption in the intestine and is indicated to aid weight loss with a reduced-calorie, low-fat diet. This drug is for adults only with obesity or overweight (BMI ≥ 27) who have risk factors like hypertension, diabetes, dyslipidemia. The over-the-counter version of orlistat (60 mg), can be taken by overweight adults with a BMI ≥ 25 (Source: www.xenical.com, www.myalli.com).

## Describing the algorithm

A major consideration for AOM prescription is BMI and the presence of weight-related comorbid conditions. The next consideration is age, as some AOM are approved for use only in adults. Fortunately, there are some AOM that could be used in children up to 12 years of age, and at least one medication for use starting at 6 years of age. The next set of considerations are based on creative matching of drug indications with corresponding need of an individual. For example, considering their dual effect on glycemic control improvement and weight loss, GLP1 receptor agonists may be the first consideration for individuals with hyperglycemia accompanying obesity. While they may have overlapping indications, AOM could also be differentiated based on their key potential to address hunger, satiety, or food cravings. Detailed questioning of patients can reveal if the main challenge is feeling unusually hungry or having difficulty feeling satiated in good time. The combination of topiramate with long-acting phentermine may be considered to address hunger, while the GLP-1 receptor agonists could be used if delayed satiety is the main issue. Another anti-obesity device expected to work by inducing satiety is hydrogel, which when consumed as a pill absorbs water and swells in the stomach, thereby reducing stomach volume and promoting fullness. The combination of naltrexone and bupropion may be beneficial if the patient has “food cravings” as a primary concern influencing food intake. Severe hunger and childhood onset of extreme obesity may suggest obesity due to specific genetic mutations, for which setmelanotide may be indicated. This medication is available for children as young as 6 years, who have the specific gene mutations. A condition to differentiate from excessive hunger is binge eating disorder. Only a subset of individuals with obesity has the disorder, characterized by episodes of consumption of enormous amounts of food and a feeling of lack of control. The phentermine/topiramate combination, has also been reported to help in addressing the binge eating disorder. If eating high fat food such as fried food poses a barrier to achieving substantial negative energy balance, orlistat may be the right AOM to reduce the digestion of fat. An inquiry into sleep hygiene and quality may lead to sleep studies and detection of obstructive sleep apnea, a condition commonly associated with obesity. The topiramate/phentermine combination is reported to produce weight loss and simultaneous improvements in obstructive sleep apnea and its associated symptoms [5].

**Fig. 1:**
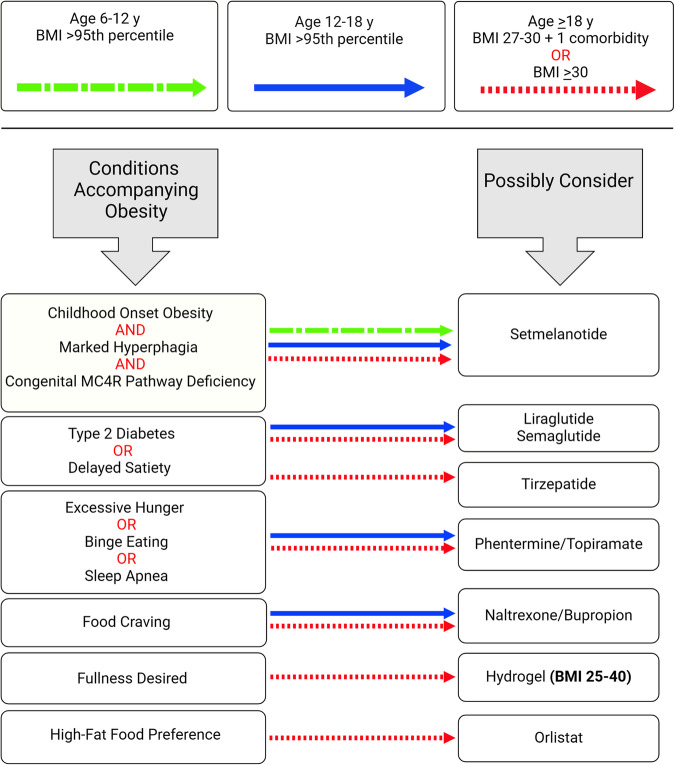
Considerations for condition-specific use of anti-obesity medications. Arrow styles indicate the BMI and age groups approved for receiving obesity medications listed below.

This algorithm focuses on currently available FDA approved AOM for long-term use (Fig. 1). As AOM, their fundamental indication is weight loss in individuals with obesity and may be used regardless of the suggested algorithm. Effective weight loss itself may alleviate many obesity-associated comorbidities without the use of an AOM with specific indication. Furthermore, clinical decisions for selecting particular AOM for an individual may be guided by medical considerations such as contra-indications or cautions for each AOM as well as non-medical considerations including cost, personal preference, and availability, which are not covered in this algorithm. However, this algorithm presents one approach to address additional needs of an individual with obesity to personalize the use of AOM, to maximize the benefits.

## References

[1] Hall KD, Kahan S (2018). Maintenance of lost weight and long-term management of obesity. Med Clin.

[2] Apovian CM, Aronne LJ, Bessesen DH, McDonnell ME, Murad MH, Pagotto U (2015). Pharmacological management of obesity: an Endocrine Society clinical practice guideline. J Clin Endocrinol Metab.

[3] Henderson K, Lewis, Sloan CE, Bessesen DH, Arterburn D (2024). Effectiveness and safety of drugs for obesity. BMJ.

[4] Daigle KM, Gang CH, Kopping MF, Gadde KM (2019). Relationship between perceptions of obesity causes and weight loss expectations among adults. J Nutr Educ Behav.

[5] Winslow DH, Bowden CH, DiDonato KP, McCullough PA (2012). A randomized, double-blind, placebo-controlled study of an oral, extended-release formulation of phentermine/topiramate for the treatment of obstructive sleep apnea in obese adults. Sleep.

